# Influence of HLA-DQ2.5 Dose on Clinical Picture of Unrelated Celiac Disease Patients

**DOI:** 10.3390/nu12123775

**Published:** 2020-12-09

**Authors:** Laura Airaksinen, Pilvi Laurikka, Heini Huhtala, Kalle Kurppa, Teea Salmi, Päivi Saavalainen, Katri Kaukinen, Katri Lindfors

**Affiliations:** 1 Celiac Disease Research Center, Faculty of Medicine and Health Technology, Tampere University, 33520 Tampere, Finland; laura.airaksinen@tuni.fi (L.A.); pilvi.laurikka@tuni.fi (P.L.); teea.salmi@tuni.fi (T.S.); katri.kaukinen@tuni.fi (K.K.); 2 Faculty of Social Sciences, Tampere University, 33520 Tampere, Finland; heini.huhtala@tuni.fi; 3 Tampere Centre for Child Health Research, Tampere University Hospital and Tampere University, 33521 Tampere, Finland; kalle.kurppa@tuni.fi; 4 Department of Pediatrics, Seinäjoki Central Hospital and University Consortium of Seinäjoki, 60220 Seinäjoki, Finland; 5 Department of Dermatology, Tampere University Hospital, 33521 Tampere, Finland; 6 Research Programs Unit, Immunobiology, and Haartman Institute, Department of Medical Genetics, University of Helsinki, 00014 Helsinki, Finland; paivi.saavalainen@helsinki.fi; 7 Department of Internal Medicine, Tampere University Hospital, 33521 Tampere, Finland

**Keywords:** celiac disease, HLA-DQ2.5, dose effect, clinical presentation, gluten-free diet

## Abstract

The clinical phenotype of celiac disease varies considerably among patients and the dosage of HLA-DQ2.5 alleles has been suggested to be a contributing factor. We investigated whether HLA-DQ2.5 allele dosage is associated with distinct clinical parameters at the time of diagnosis and with patients’ response to a gluten-free diet. The final cohort included 605 carefully phenotyped non-related Finnish celiac disease patients grouped as having 0, 1 or 2 copies of HLA-DQ2.5. Clinical data at the time of diagnosis and during gluten-free diet were collected systematically from medical records and supplementary interviews. An increasing HLA-DQ2.5 dose effect was detected for celiac disease antibody positivity at diagnosis (*p* = 0.021) and for the presence of any first-degree relatives with celiac disease (*p* = 0.011 and *p* = 0.031, respectively). Instead, DQ2.5-negative patients were suffering most often from classical symptoms at diagnosis (*p* = 0.007 between HLA groups). In addition, during follow-up they were most often symptomatic despite a gluten-free diet (*p* = 0.002 between groups). Our results thus suggest that increasing HLA-DQ2.5 dose only has a minor effect on the clinical picture of celiac disease. However, HLA-DQ2.5-negative patients should not be overlooked in clinical practice and particular attention should be paid to this patient group during gluten-free diet.

## 1. Introduction

Celiac disease is a chronic immune mediated condition driven by the ingestion of dietary gluten. It is characterized by small bowel mucosal damage and autoantibody response to transglutaminase 2 (TG2). The disease can be diagnosed at any age from childhood to older age and there is often a marked diagnostic delay [1,2]. Moreover, there is a substantial variation in the clinical picture of the disease, which may present with gastrointestinal and/or extraintestinal symptoms of varying severity or be completely asymptomatic [3]. Further variation to the disease phenotype is brought by several associated conditions [4]. The only efficient treatment for celiac disease is a strict gluten-free diet (GFD), which usually results in alleviation of symptoms and normalization of small bowel mucosal morphology. Nevertheless, in a subset of patients, the symptoms and mucosal damage may persist despite a GFD [5,6]. If malabsorption and villous atrophy persist despite strict avoidance of gluten for a minimum of 12 months on GFD and after alternative causes have been excluded, the condition is termed refractory celiac disease (RCD) [7].

Family members of celiac disease patients are at an increased risk of being affected, likely due to their higher frequency of HLA-DQ2.5 or HLA-DQ8, the major determinants contributing to disease susceptibility [8]. HLA-DQ2.5 and DQ8 are heterodimeric molecules present on the surface of antigen presenting cells. In celiac disease, they bind and present deamidated gluten peptides to CD4-positive T cells, leading to the generation of an immune response [9,10]. HLA-DQ2.5 is far more common than HLA-DQ8 as it is present in more than 90% of patients [11]. The HLA-DQ2.5 heterodimer can be encoded by HLA-DQA1*0501 and HLA-DQB1*0201 alleles located on the same chromosome (*cis*) or by DQA1*0505 and DQB1*0202 on different chromosomes (*trans*). Homozygosity for HLA-DQ2.5 is associated with a particularly high risk for celiac disease [12,13,14]. This phenomenon has been attributed to the premise that gluten presented by antigen presenting cells in HLA-DQ2.5 homozygous individuals can induce a four-fold higher T cell response than in heterozygous individuals [15]. In addition to the disease risk, the dose of HLA-DQ2.5 has been suggested to affect the phenotype of celiac disease [16]. Earlier research on this issue has nevertheless reported conflicting findings, possibly due to rather small patient cohorts comprising a substantial portion of pediatric patients [16]. Nowadays, the majority of celiac disease diagnoses are made on adults and their clinical picture may be contributed to a larger extent to factors other than the HLA-type. Moreover, earlier research has not excluded patients originating from the same family, thereby increasing the possibility of bias caused by similar genetic background.

We investigated the HLA-DQ2.5 dose effect on various clinical parameters at the time of diagnosis and on patients’ response to GFD by exploiting a large and carefully phenotyped cohort of unrelated pediatric and adult celiac disease patients.

## 2. Materials and Methods

### 2.1. Patients and Study Design

The study was conducted at Tampere University and Tampere University Hospital. Altogether, 1048 biopsy-proven celiac disease patients were recruited by a nationwide search with the help of national and local celiac societies and by media announcements. The patient information at diagnosis (demographics, clinical and histological data, celiac disease serology, presence of symptoms during childhood, and other concomitant chronic medical conditions as well as family history of celiac disease) was collected from medical records and from supplementary interviews by a physician or a study nurse. In the case of children, the guardian was interviewed. Patients were divided into four different age groups: 0–6 years, 7–20 years, 21–65 years, and >65 years. Diagnostic delay was categorized as 0 (screen-detected patients), <1, 1–5, 5–10 or >10 years. Abdominal symptoms included abdominal pain, diarrhea, loose stools, heartburn, flatulence, constipation, and/or bloating. Malabsorption was defined as weight loss and/or presence of characteristic laboratory abnormalities, such as anemia, hypoalbuminemia, low folate or low vitamin B12. Classical symptoms referred to the presence of both diarrhea and malabsorption [17]. Extraintestinal symptoms included any symptom(s) presenting outside of the gastrointestinal tract, such as dermatitis herpetiformis, infertility, joint pains, and neurological problems. Severity of symptoms was categorized into “no symptoms”, “mild symptoms”, “moderate symptoms” or “severe symptoms”. “First-degree relative” referred to sibling, mother, father or offspring. “Relative” referred to any relative in a family. In addition, follow-up data on self-reported current symptoms, both gastrointestinal and extraintestinal, as well as adherence to GFD, were assessed by interviews. Adherence to GFD was described as either “strict GFD”, “dietary lapses” or “no GFD”. In addition, histological and serological data at follow-up were assessed as described below.

In order to avoid false positive findings due to trait correlation between genetically related individuals, only one patient from each family was included (randomly). The final study cohort included 605 celiac disease patients.

The study design, patient recruitment, and collection of patient record data were approved by the Regional Ethics Committee of Tampere University Hospital. All participants gave written informed consent.

### 2.2. Histology

The results of histological analysis of the small-bowel mucosal biopsies at the time of diagnosis were collected from the pathology reports. In addition, if available, the degree of mucosal recovery on GFD evaluated from possible repeat biopsy was recorded. In both cases, severity of small intestinal mucosal damage was evaluated from several representative and well-orientated biopsy specimens and the degree of diagnostic villous atrophy was classified as partial, subtotal, or total, corresponding approximately to the IIIa, IIIb, and IIIc Marsh–Oberhuber classifications, respectively [18].

### 2.3. Serology

The results of celiac disease serology at the time of diagnosis were collected from the medical records. A patient was regarded as positive for celiac disease-specific antibodies if he or she was positive for TG2 autoantibodies [19] and/or endomysial autoantibodies (EmA) [20] and/or antireticulin autoantibodies (ARAs, measured in 1980/90s and later replaced by EmA) [21]. ARA and EmA were analyzed using indirect immunofluorescence with rat liver, kidney or stomach tissue (ARA) [21] or human umbilical cord (EmA) [22] as an antigen. Titers 1: ≥5 were considered positive. From serum samples collected at the time of the present study (follow-up data), TG2 antibodies and EmA were determined. TG2 antibodies values were tested by enzyme-linked immunosorbent assay (QUANTA Lite h-tTG IgA, INOVA Diagnostics, San Diego, CA, USA) with the cut-off for positivity being >30 U/L.

### 2.4. Genetic Analysis

The genotypes corresponding to disease-associated HLA variants, HLA-DQ2.5, HLA-DQ8, and HLA-DQ2.2, were determined using commercial HLA typing kits (Olerup SSP low-resolution kit, Olerup SSP AB, Saltsjöbaden, Sweden or DELFIA^®^ Celiac Disease Hybridization Assay Kit, PerkinElmer Life and Analytical Sciences, Wallac Oy, Turku, Finland) or the TaqMan chemistry based genotyping of the HLA tagging single-nucleotide polymorphisms (SNPs) as previously described [23,24]. In this study, patients carrying alleles HLA-DQB1*0201 and HLA-DQA1*0501 in *cis* configuration were considered positive for HLA-DQ2.5. The subjects were divided into three groups according to whether they had zero, one or two copies of HLA-DQ2.5.

### 2.5. Statistics

Statistical analyses were performed with SPSS Statistics version 23 (IBM Corp, Armonk, NY, USA). Variables were presented as percentages and tested by Chi-square test or Fisher’s Exact test, as appropriate. *p* value < 0.05 was considered significant across all analyses. Statistical analyses were performed for all patients together and for younger patients (<21 years, *n* = 124) and adults (*n* = 476) separately.

## 3. Results

Altogether, 100 (16.5%) celiac disease patients were negative (X/X group), 401 (66.3%) were heterozygous (DQ2.5/X group), and 104 (17.2%) were homozygous (DQ2.5/DQ2.5 group) for HLA-DQ2.5 (Table 1).

**Figure 1:**
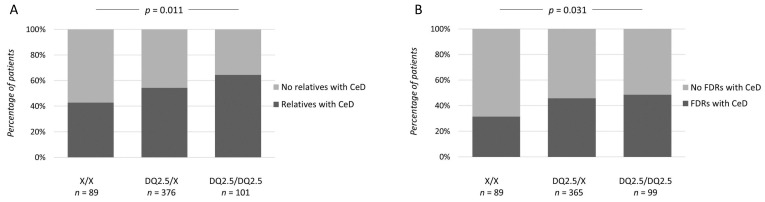
The percentages of patients among different HLA-DQ2.5 dose groups having (**A**) any relatives with CeD and (**B**) having first-degree relatives (FDR) with CeD. Patients are divided into different HLA-DQ2.5 dose groups based on whether they are either negative (X/X), heterozygous (DQ2.5/X) or homozygous (DQ2.5/DQ2.5) for HLA-DQ2.5.

At the time of diagnosis, there was no significant difference in age distribution between the HLA-DQ2.5 dose groups. The proportion of patients suffering from classical symptoms was lowest among heterozygotes and highest among DQ2.5-negative patients (Table 1). No significant differences between the groups were observed in presence of symptoms in childhood, diagnostic delay or the type (abdominal, diarrhea, anemia, extraintestinal) or severity of symptoms (Table 1). The percentage of patients positive for celiac disease-specific autoantibodies was smallest in the HLA-DQ2.5-negative group and greatest in HLA-DQ2.5-homozygous group. The groups were comparable in terms of severity of mucosal damage (Table 1). DQ2.5 homozygotes most often had any relative or a first-degree relative with celiac disease, whereas DQ2.5-negative patients had such relatives the least often (Figure 1A,B).

At follow-up (median follow-up time 13 years, range <1–47 years), the DQ2.5-heterozygous patients maintained a strict GFD most often and homozygous patients least often (Table 2). The proportion of patients suffering from self-reported current symptoms was greatest among DQ2.5-negative and smallest among DQ2.5-heterozygous patients. Groups did not differ significantly in terms of antibody positivity, mucosal recovery, concomitant autoimmune diseases or malignancy at the time of the follow-up (Table 2).

The results of a subanalysis with adult patients only were parallel to those of the whole cohort. Significant differences between distinct HLA-DQ2.5 dose groups were observed in the presence of classical symptoms and celiac disease-specific antibody positivity at diagnosis (*p* = 0.006 and *p* = 0.043, respectively). Moreover, HLA-DQ2.5 homozygous adults also had more often either any relative or a first-degree relative with celiac disease than did heterozygous or HLA-DQ2.5-negative patients (*p* = 0.001 for any relative and *p* = 0.003 for first-degree relative). For adults at follow-up, significant differences between dose groups were observed in adherence to the GFD and in self-reported current symptoms (*p* = 0.016 and *p* = 0.002, respectively). When children were analyzed separately, no significant differences were found in any of the parameters studied (data not shown).

## 4. Discussion

It has previously been reported that HLA-DQ2.5 dose is associated with increased risk of celiac disease [14], stronger disease-specific T cell response in vitro [15], and the presence of classical symptoms, particularly in children [16,25]. Here, we observed a dose effect for celiac autoantibody positivity at diagnosis as well as for the presence of any and first-degree relatives with the disease. However, we observed no increase in the presence of classical symptoms with an increasing HLA-DQ2.5 dose; instead, the HLA-DQ2.5-negative group presented most often with this phenotype. In addition, we found no association between HLA-DQ2.5 dose and abdominal symptoms, diarrhea, anemia, extraintestinal manifestations, severity of symptoms or mucosal morphology.

Our finding of an HLA-DQ2.5 dose effect with antibody positivity is rational since the gluten-specific T cells that proliferate and activate more robustly after stimulation in the context of the homozygous HLA-DQ2.5 antigen presenting cells participate in the induction of an anti-TG2 antibody response [26]. Moreover, such a dose effect in patients having a relative with celiac disease likely reflects the presence of the predisposing HLA-DQ2.5 within these families. When comparing our results with those of earlier studies, it is noteworthy that our cohort included mostly adult celiac disease patients, whereas earlier studies were conducted predominantly on pediatric patients [16]. It is possible that the factors affecting the disease phenotype differ between adults and children and also include other determinants besides the HLA-DQ type. The identity of such phenotype-modulating factors remains obscure but may, for instance, include non-HLA genetic variants and/or environmental factors. This assumption is supported by our previous finding that sib pairs with discordant clinical presentation had similar HLA haplotypes more often than pairs with the concordant phenotype did [27]. Interestingly, we have also observed that the diversity and composition of intestinal microbiota varies markedly between different celiac disease phenotypes, this being an interesting issue for further study [28].

HLA-DQ2.5 homozygosity has previously been observed in over 40% of patients with RCD type II (RCDII) in contrast to 20% in uncomplicated celiac disease [29]. RCDII is a severe condition with a poor prognosis [7]. Due to this and the fact that RCDII is very rare in Finland [30], our cohort did not include such cases and we were unable to address the HLA-DQ2.5 dose effect on this parameter. In any case, we found that HLA-DQ2.5 dose is not associated with poorer recovery of the intestinal damage. The investigation of HLA-DQ2.5 dose in RCDII would require a multi-center approach to achieve a sufficient number of patients for statistical power.

We found patients negative for HLA-DQ2.5 to suffer most often from classical symptoms at celiac disease diagnosis. Moreover, they most often experienced symptoms during GFD although their adherence to GFD was excellent. In addition, at follow-up, they did not differ from the other HLA-DQ2.5 groups in terms of celiac disease antibody positivity or small bowel mucosal morphology. A long diagnostic delay has been reported to predispose to persistent symptoms [31], but here patients negative for HLA-DQ2.5 received their diagnoses within the same time limits as the other HLA groups. Alternative explanations for persistent symptoms while on GFD could be altered composition of the small bowel mucosal microbiome [32], small-intestinal bacterial overgrowth [33] or continuous low-grade inflammation in spite of a strict GFD [34]. In any case, our results stress the need to pay special attention to HLA-DQ2.5-negative patients in clinics in order to prevent long-lasting health problems in this patient subset.

### 4.1. Strengths and Weaknesses

The main strength of our study was a large and well-defined cohort of unrelated adult and pediatric celiac disease patients enabling us to address the effect of HLA-DQ2.5 dose reliably. However, the study was retrospective, which, given its nature may appear as a limitation. In addition, our study considered HLA-DQ2.5 alleles only in the *cis* configuration. Therefore, HLA-DQ2.5/HLA-DQ2.2 genotype was categorized as HLA-DQ2.5-heterozygous in spite of evidence to suggest that this genotype carries an equal risk for celiac disease as HLA-DQ2.5 homozygosity [35]. Moreover, the HLA-DQ2.5-negative group was heterogenous, comprising both the HLA-DQ8-positive cases as well as those without any of the major HLA types predisposing to celiac disease. Further, the small number of pediatric patients inhibited any reliable investigation of the effect of HLA-DQ2.5 dose in this particular subgroup.

### 4.2. Conclusions

In our cohort with a preponderance of adults, we demonstrated that the effect of HLA-DQ2.5 dose on the clinical picture of celiac disease was only modest. Patients negative for HLA-DQ2.5 were characterized by the most marked seronegativity, by the presence of classic symptoms at diagnosis, and also by symptoms persisting in spite of GFD.

## Figures and Tables

**Table 1:** Clinical, serological and histological characteristics of 605 celiac disease patients negative (X/X), heterozygous (DQ2.5/X) or homozygous (DQ2.5/DQ2.5) for HLA-DQ2.5 at the time of diagnosis.

	X/X	DQ2.5/X	DQ2.5/DQ2.5	*p*
	*n* = 100	*n* = 401	*n* = 104	
	% (*n*)	% (*n*)	% (*n*)	
Females	78.0 (78)	73.3 (294)	76.0 (79)	0.589
Age group				0.996 *
0–6 years	7.1 (7)	6.8 (27)	7.7 (8)	
7–20 years	13.1 (13)	13.6 (54)	14.4 (15)	
21–65 years	75.8 (75)	76.1 (302)	73.1 (76)	
>65 years	4.0 (4)	3.5 (14)	4.8 (5)	
Symptoms in childhood	48.9 (43)	43.8 (161)	51.5 (51)	0.334
Diagnostic delay				0.908 *
0 ^1^	4.3 (4)	4.9 (18)	3.0 (3)	
<1 year	20.7 (19)	23.6 (86)	25.7 (26)	
1–5 years	37.0 (34)	31.5 (115)	32.7 (33)	
5–10 years	7.6 (7)	10.4 (38)	6.9 (7)	
>10 years	30.4 (28)	29.6 (108)	31.7 (32)	
Classical symptoms ^2^	30.0 (30)	16.7 (67)	21.2 (22)	**0.007**
Abdominal symptoms ^3^	86.9 (86)	81.3 (322)	81.7 (85)	0.426
Diarrhea	44.7 (42)	37.1 (144)	41.7 (43)	0.337
Anemia	30.3 (30)	28.5 (113)	37.5 (39)	0.209
Extraintestinal manifestations ^4^	44.4 (44)	49.5 (196)	47.1 (49)	0.646
Dermatitis herpetiformis	11.0 (10)	17.1 (63)	19.2 (19)	0.270
Severity of symptoms ^5^				0.484 *
No symptoms	5.0 (4)	5.9 (17)	4.0 (3)	
Mild	23.8 (19)	25.9 (74)	33.3 (25)	
Moderate	15.0 (12)	12.2 (35)	5.3 (4)	
Severe	56.3 (45)	55.9 (160)	57.3 (43)	
Celiac disease antibody positivity ^6,7^	77.8 (49)	86.8 (217)	94.9 (56)	**0.021**
Severity of mucosal damage ^8^				0.546 *
Normal morphology	2.4 (2)	3.0 (10)	2.4 (2)	
Partial villous atrophy	27.7 (23)	33.8 (111)	25.3 (21)	
Subtotal/total villous atrophy	69.9 (58)	63.1 (207)	72.3 (60)	

Values in bold face indicate statistically significant difference with *p* value < 0.05. * Calculated across all variables. Data were available for >90% of patients except for designated parameters where number of cases in different HLA-DQ2.5 dose groups was ^5^ 80, 286, 75; ^7^ 63, 250, 59 and ^8^ 83, 328, 83, respectively. ^1^ Screen-detected patients. ^2^ Presence of diarrhea and malabsorption. ^3^ Abdominal pain, diarrhea, loose stools, heartburn, flatulence, constipation, and/or bloating. ^4^ Any symptom(s) presenting outside of the gastrointestinal tract, such as dermatitis herpetiformis, infertility, joint pains and neurological problems. ^6^ Positivity for serum TG2 autoantibodies and/or endomysial autoantibodies and/or antireticulin autoantibodies.

**Table 2:** Clinical, serological and histological characteristics of 605 celiac disease patients negative (X/X), heterozygous (DQ2.5/X) or homozygous (DQ2.5/DQ2.5) for HLA-DQ2.5 at the time of the follow-up.

	X/X	DQ2.5/X	DQ2.5/DQ2.5	
	*n* = 100	*n* = 401	*n* = 104	
	% (*n*)	% (*n*)	% (*n*)	*p*
Adherence to GFD ^1^				0.025 *
Strict GFD ^1^	93.8 (91)	97.4 (376)	91.1 (92)	
Dietary lapses	5.2 (5)	2.3 (9)	7.9 (8)	
No GFD ^1^	1.0 (1)	0.3 (1)	1.0 (1)	
Self-reported current symptoms ^2,3^	44.8 (26)	22.9 (59)	32.3 (20)	**0.002**
Celiac disease antibody positivity ^4,5^	28.6 (4)	23.8 (10)	9.1 (1)	0.535
Severity of mucosal damage ^6^				0.108 *
Normal morphology	72.9 (35)	55.6 (104)	46.9 (23)	
Partial villous atrophy	25.0 (12)	38.0 (71)	44.9 (22)	
Subtotal/total villous atrophy	2.1 (1)	6.4 (12)	8.2 (4)	
Other illnesses				
Any autoimmune disease ^7^	29.8 (28)	23.6 (91)	18.0 (18)	0.155
Type 1 diabetes	3.2 (3)	3.4 (13)	1.0 (1)	0.504
Thyroidal disease	17.9 (17)	14.0 (54)	9.7 (10)	0.248
Malignancy	3.2 (3)	3.9 (15)	5.8 (6)	0.581

Values in bold face indicate statistically significant difference with *p* value < 0.05. * Calculated across all variables. Data were available for >90% of patients except for designated parameters where numbers of cases in different HLA-DQ2.5 dose groups were ^3^ 58, 258, 62; ^5^ 14, 42, 11 and ^6^ 48, 187, 49, respectively. ^1^ GFD = gluten-free diet. ^2^ Any type of recurrent gastrointestinal and extraintestinal symptoms. ^4^ Positivity for serum TG2 and/or endomysial and/or antireticulin autoantibodies. ^7^ Any autoimmune disease including type 1 diabetes (DM1), thyroidal diseases, IgA nephropathy, Sjögren’s syndrome, rheumatoid arthritis, sarcoidosis, psoriasis, vitiligo, and lichen planus.
